# Hints for Working Celluloid

**Published:** 1877-11

**Authors:** Edgar Palmer

**Affiliations:** Lacrosse, Wis.


### HINTS FOR WORKING CELLULOID.

BY EDGAR PALMER., D. D. S., LACROSSE, WIS.

Having passed through the several stages so familiar to those who attempt the use of new methods and materials, experiencing alternate success and failure in the working of celluloid, and having settled upon a special method, I offer it to any who are unsuccessful in its use, not as something novel or original, but simply as the method affording me, under a trial of several months, the most uniform and gratifying results.

1. To overcome the breaking and defacing of models: After taking the impression with plaster alone in the ordinary way, dry it thoroughly, wind around it a paper, and pour a model of pure tin, and upon this make your plate. Keep the face of your model well coated with powdered soap stone to prevent wax and the celluloid from sticking to it, and the plate will come out of the press not only clean and perfect, but polished on the palatine surface.

The breaking of teeth is done usually by insufficient heat, and the fact that they are waxed too low down on the teeth before investment.

The warping and springing of plates to the carelessness and haste in working the material. Give the plates at least fifteen hours to cure in the press, and this trouble will have been overcome. Use "dry heat," and if you have the old steam boiler, go to any machinest and get him to drill eighteen holes, three eighths of an inch in diameter, through the bottom. This will bring three between each rib. Cut out round a heavy piece of sheet iron, three and one half inches in diameter, and lay on the ribs. Take off your collar and packing from the top where the screw enters, and remove the lead weight from the steam escape and lay them aside. Enlarge the hole and insert a therometer from where you removed the weight, securing it with plaster; and at an outlay of about $1.75 you have an oven equal to any in the market. And if you are of the economical order, save the cost of alcohol by using the "Summer Queen" oil stove. I use a No. 2, and like it much better than alcohol.

I am one of the advocates of celluloid, and am more ready to attribute faulty plates to my own unskillfulness in working it than to condemn the material because it fails, in some cases, to fulfill my expectations.

